# Towards Automated Spine Fracture Detection on Whole-Body CT of Polytraumatized Patients

**DOI:** 10.3390/jimaging12060265

**Published:** 2026-06-18

**Authors:** Elena Stojanovski, Alexander Hönning, Frederik Spohn, Marlene Ciesla, Holger Arndt, Sven Mutze, Alena-Kathrin Golla, Tobias Klinder, Cristian Lorenz, Leonie Goelz

**Affiliations:** 1 Institute of Radiology and Neuroradiology, BG Klinikum Unfallkrankenhaus Berlin, 12683 Berlin, Germany; 2 Center for Clinical Research, BG Klinikum Unfallkrankenhaus Berlin, 12683 Berlin, Germany; 3 Institute of Diagnostic Radiology and Neuroradiology, Universitätsklinikum Greifswald, 17489 Greifswald, Germany; 4 Philips Research Hamburg, Roentgenstrasse 24-26, 22335 Hamburg, Germany

**Keywords:** whole-body CT, spine fracture, traumatic, automated detection, AI

## Abstract

Treatment of severely injured patients is challenging, and timely reading of whole-body computed tomography (WBCT) images therefore crucial. Artificial intelligence is increasingly used to prioritize and detect acute injuries in this context. Algorithms focusing on the cervical spine and compression fractures have been deployed successfully. However, tools for whole spine assessment and the entirety of fracture morphologies are lacking. We aimed to investigate the capabilities of an algorithm to detect spine fractures on WBCTs and factors contributing to the difficulties in its development. A version 1.0 (v1) of the algorithm was previously trained with 454 cervical spine fractures using a U-Net via four-fold cross-validation to segment spine fractures and the spine via a multi-task loss. Further training expanded towards whole spine assessment with additional annotated fractures (Cohort 1) of the cervical (*n* = 50), thoracic (*n* = 30), and lumbar spine (*n* = 20), resulting in version 2.0 (v2). Baseline was set to reach the highest sensitivity at a maximum of five false positives per case. Version 1.0 was tested on Cohort 1 and both versions were compared on prospectively collected real-world data (Cohort 2, *n* = 712 WBCTs). An additional systematic review served to compare the algorithmic performance against the state-of-the-art. Version 1.0 showed promising performance not only for the cervical but also the thoracic and lumbar spine due to generalization (sensitivities ranging between 60% and 87%). Version 2.0 also achieved decent sensitivities for Cohort 2 (sensitivities ranging between 77% and 85%) but generated an abundance of false positives. Various reasons led to false positive results; for Version 2.0, the trabecular structure itself provoked false alerts. Variances in training and test data (image quality, dose, reconstructions), heterogeneity of fractures and anatomies, plus the size of training sets explain some difficulties during algorithm development. Only five other groups described their work on whole-spine fracture detection, encountered similar difficulties, and have also failed to develop a clinically deployable tool. Spine fracture detection on WBCT is feasible, but multiple factors hinder the development of commercially available AI tools. Expansion and the improved design of training cohorts are necessary for further development and simulation of real-life conditions.

## 1. Introduction

Treatment of severely injured patients in the emergency department (ED) can be a challenging task for doctors around the world [1]. During trauma care, comprehensive assessment of all critical injuries through whole-body computed tomography (WBCT) has been established as a tool for clinical decision-making, surgical planning, and triaging of multiple injuries [2]. The relevance of speed during radiological workup is reflected by measurements of the “time to trauma CT” [3] and efforts to establish maximum proximity between trauma room and CT scanner [4]. Obviously, improvement of trauma workflows cannot be achieved merely by performing WBCT as quickly as possible. Accelerating the completion of concise radiology reports is equally crucial. Therefore, research has been directed toward structured reporting of WBCT [5] and implementation of artificial intelligence (AI) in emergency radiology and emergency medicine as a whole [6].

Today, radiologists, neurosurgeons, trauma, and orthopedic surgeons are supported by triage, detection, and quantification tools for acute injuries such as intracranial hemorrhages, rib fractures, pneumothorax, abdominal free gas, and fluid [7,8]. However, spine fractures with a delayed diagnosis are one cause of increased morbidity in severely injured patients, especially for cervical spine fractures and thoracolumbar fractures [9,10]. Recently, several deep learning algorithms for cervical spine fracture detection have been proposed, notably further accelerated via a dedicated challenge initiate by the Radiological Society of North America in 2022 [11]. Other researchers have developed approaches to detect vertebral compression fractures, many of which focus on incidental findings in the context of osteoporosis rather than acute trauma [12]. While cervical spine and compression fractures also occur in severe trauma, available approaches do not address other potentially unstable traumatic spine fractures, particularly those involving the dorsal aspects of the vertebrae, which are critical for spinal stability and clinical decision-making.

WBCT assessment under time pressure is especially challenging because a number of critical injuries have to be excluded quickly. The initial survey by trauma surgeons is instantly proceeded by transfer to the nearby CT scanner. WBCT-centered management of severely injured patients is specified in international trauma guidelines after proof of its positive impact on patient outcomes [13]. However, rather little progress has been achieved over the last years to develop a more comprehensive automated fracture assessment of the whole spine.

Building on our earlier work on automated cervical spine fractures [14], we extend the approach of identifying each fracture line to cover the entire spine of trauma patients. For that purpose, the algorithm was further trained with additional data covering the thoracic and lumbar spine region. Studying the behavior of AI tools in real-world settings is resource-intensive but crucial to determine their applicability. Interdisciplinary research between physicians and developers is key to identify and address pain points of clinical care. Therefore, we sought to provide real-life data on investigating the capabilities of an algorithm to detect all types of spine fractures on WBCTs of severely injured patients. We then aimed to analyze factors contributing to the difficulties in the development of such algorithms through an additional systematic review.

## 2. Materials and Methods

### 2.1. Patient Cohorts

Cohort 1 comprised patients with a prevalence of 100% for spine fractures: 100 consecutive patients with confirmed acute spine fractures on CT imaging (50 cervical, 30 thoracic, and 20 lumbar fractures) were identified through the hospital information system of an academic metropolitan trauma center in Germany between July 2017 through December 2020 and included retrospectively. Images with and without contrast enhancement were acquired using a 2 × 192 row dual-source scanner (Somatom Force, Siemens, Erlangen, Germany). Cohort 1 served as an additional training set for the detection model v2, and was used for an assessment of the performance of model v1 on the extended set of fracture locations as a proof of concept before expansion of the clinical cohort.

**Figure 1:**
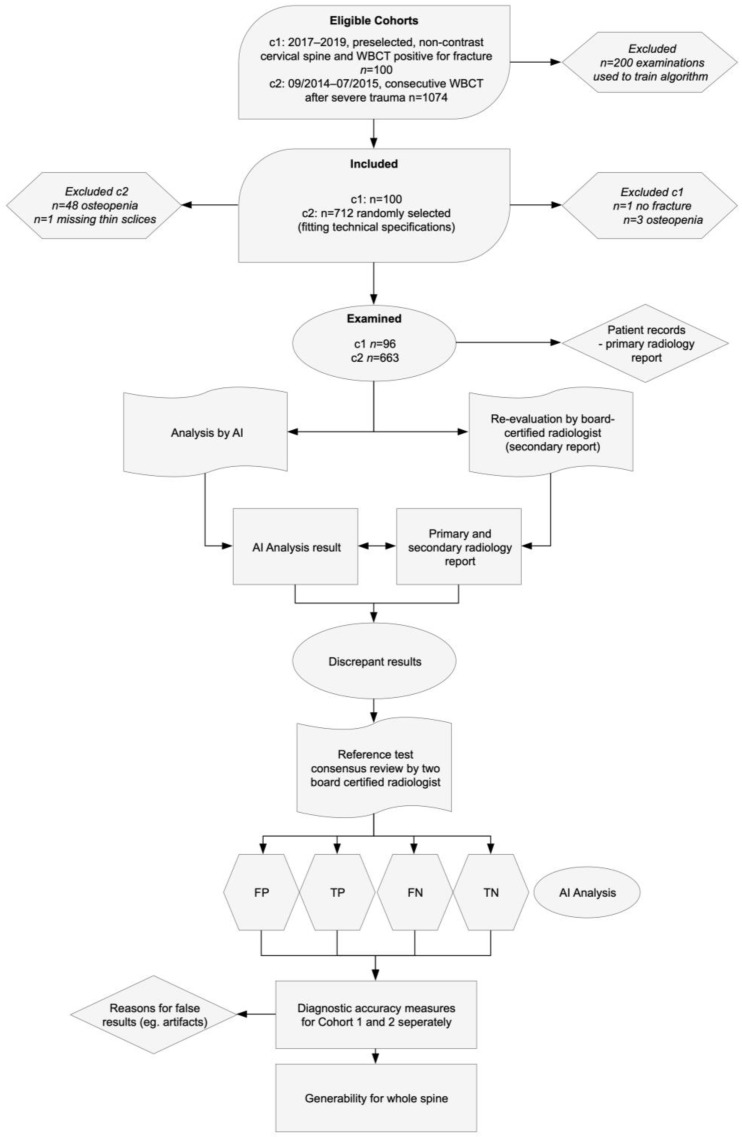
Flow diagram of study conception, screening, inclusion, and exclusion of individuals (c1—Cohort 1, c2—Cohort2, WBCT—whole-body CT, AI—artificial intelligence, FP—false positive, TP—true positive, FN—false negative, TN—true negative).

Cohort 2 comprised real-world data of severely injured patients examined using a 128-row scanner (Philips Ingenuity Core, Philips Healthcare, Amsterdam, The Netherlands). Contrast-enhanced WBCTs were performed with and without the use of iterative and filtered backprojection reconstructions for dose reduction (iDose^®^, Philips Healthcare, Amsterdam, The Netherlands). The prevalence of spine fractures in this cohort was not analyzed explicitly. In a secondary analysis of the DOREMI trial [15], 1074 prospectively enrolled consecutive patients admitted for suspected multiple traumata between September 2014 and August 2016 were eligible. Cases were randomly selected and processed until the maximum logging capacity of hard- and software were reached (Figure 1).

The following study was registered prospectively at the German Registry for Clinical Studies (DRKS: DRKS00029356) and conducted in accordance with the Declaration of Helsinki in its current form. The review of Charité Universitätsmedizin Berlin (EA2/048/13) approved the DOREMI trial in 2013 and participants provided written informed consent. The Medical Association of Berlin, Germany (Eth-23/22), approved the protocol of the current analysis and waived the need for written consent.

### 2.2. AI Algorithm

In a scientific collaboration, an algorithm for the detection of spine fractures was developed between 2018 and 2023. A first Version 1.0 (v1) of the algorithm was trained with 195 CTs (cervical and WBCTs) of 454 cervical spine fractures including vertebral body, vertebral arch, spinous process, lateral process, articulate process, odontoid (C2), and lateral mass (C1). Four radiologists with 2–7 years of experience in emergency radiology annotated the images manually in a consensus fashion on axial, sagittal, and coronal planes in Microsoft Word (Version 2016, Microsoft, Redmond, WA, USA) and exported the anonymized images with an in-plane and through-plane resolution (min—average—max) of 0.25—0.73—0.98 mm and 0.4—0.62—0.7 mm, respectively, from the PACS (Philips IntelliSpace 4.4, Philips, Eindhoven, The Netherlands). Imaging was obtained using a 2 × 192 row dual-source scanner (Somatom Force, Siemens, Erlangen, Germany).

A U-Net via 4-fold cross-validation was trained to perform a vertebra-wise classification as a segmentation task. The best detection rate was achieved by processing the data using spinal-canal-aligned volumes of interest. During training, data augmentation was performed to expand the training dataset. Version 1.0 detected 87.2% of cervical spine fractures at an average number of false positives of 3.5 per case. The technical details and its performance have been published previously [14]. Of note, v1 was able to segment the whole spine and showed the ability to detect fractures of the thoracic and lumbar spine contained in the training and validation data which had not been annotated.

Motivated by this performance, further training was performed in the described fashion using an additional 100 CT images (Cohort 1) with annotated fractures of all structures (vertebral body, vertebral arch, spinous process, lateral process, articulate process) of the cervical (*n* = 50), thoracic (*n* = 30), and lumbar spine (*n* = 20), resulting in Version 2.0 (v2). As such, v2 aimed at explicitly expanding towards whole-body assessment of the spine.

After the described training steps, both algorithms were set to reach the highest sensitivity at a maximum of five false positives per case for the proof of concept as well as for the expanded clinical study.

### 2.3. AI Analysis

The algorithm was deployed on a Lenovo ThinkPad (11th Gen Intel^®^ Core™ i9-11950H @ 2.60 GHz 2.61 GHz) at the trauma center within the hospital’s network under Windows 10 Enterprise (Microsoft, Redmond, WA, USA). Cohort 1 served to test v1 on the extended set of fracture locations. The first hundred cases of Cohort 2 were used to pilot-test v2 versus the earlier developed v1. This was followed by dedicated cohort testing of v2 with all cases of cohort 2.

**Figure 2:**
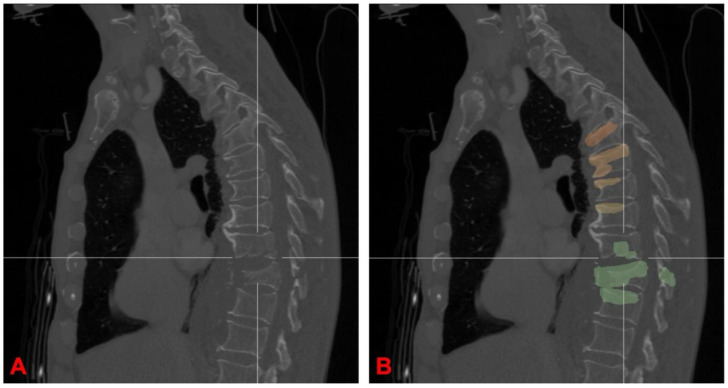
True positive analysis by the AI prototype v1 showing an osseous disruption of the mid-thoracic spine and three impression fractures (**A**). Highlighted findings (**B**) of the analysis, with detection of traumatic impression fractures of the upper thoracic spine (brown shades) and osseous disruption of the mid-thoracic spine (green).

The analysis was performed in four steps: export of the CTs from the PACS onto the laptop, preprocessing of the CTs by the prototype, analysis for spine fractures, and display of findings (Figure 2).

### 2.4. Human Readers and Discrepancy Analysis

CT images were read by a junior radiologist with 1–5 years of experience in emergency radiology and a senior radiologist with >10 years of experience in emergency radiology. Fractures were classified by level, segment of the spine, and anatomical structure.

A third independent radiologist with 4 years of experience in emergency radiology exclusively searched for spine fractures in both cohorts and compared these results with the findings of the prototype. Discrepancies were resolved by a neuroradiologist. Additionally, possible reasons for false AI analysis results were recorded.

### 2.5. Statistical Analysis

Results of AI analysis were classified into true positive (TP), true negative (TN), false positive (FP), and false negative (FN) per WBCT scan and per fracture. The primary endpoint was the ability of the algorithm to detect spine fractures after segmentation of WBCTs, determined as sensitivity (TP/[TP + FN]) with 95% Clopper-Pearson confidence intervals (CIs). Sensitivities were reported per WBCT scan and per fracture. As specificity was unsuitable to assess the algorithm’s clinical usability with the selected and above-described working settings, false positives per case were recorded from 0 to 3 and as ≥3.

As a secondary endpoint, we determined the performance of the algorithm for each spinal segment. Clinical parameters and impact were evaluated using descriptive statistics including arithmetic mean, standard deviation (SD), minimum and maximum values (range), and absolute (*n*) and relative (%) proportions. All statistical analyses were performed using STATA for Windows (version 16.1).

### 2.6. Systematic Database Search

**Figure 3:**
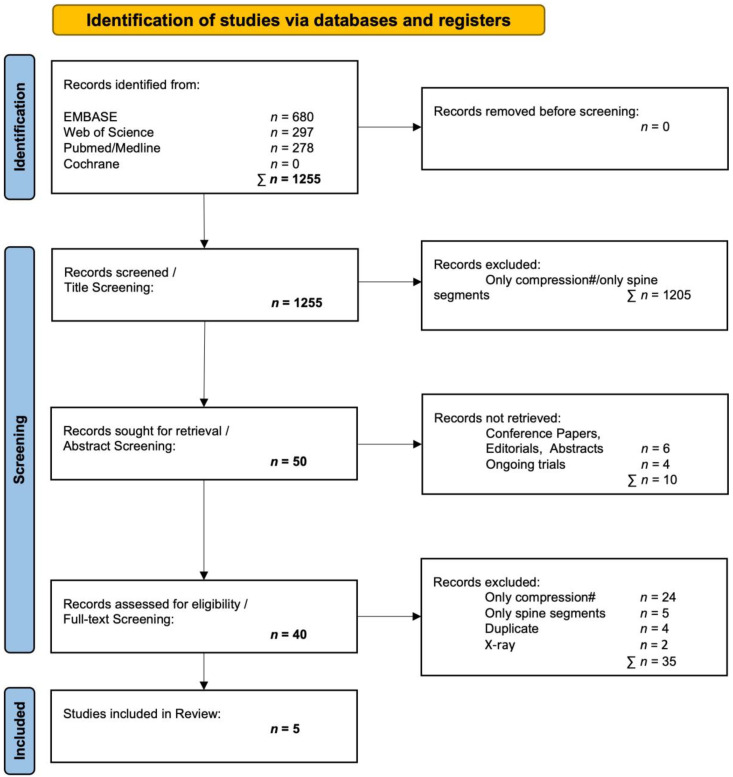
PRISMA Flow Chart 2020 adapted from Page MJ et al., 2021 [16].

In order to compare our algorithmic performance against the state-of-the-art, we conducted a systematic review. Two radiologists with 5 and 11 years of training searched EMBASE, Web of Science, Pubmed/Medline, and Cochrane independently for studies about AI solutions designed to detect traumatic fractures of all levels of the spine on CT imaging according to the PRISMA Guidelines [16] in February 2026 (see Table S1 for search strategies). Divergent results were solved by consensus. Articles describing algorithms which exclusively detect compression fractures (often in the context of osteoporosis) or fractures at a defined spine segment were excluded (Figure 3). Suitable full-text articles in English published between 2015 and 2025 were included in the study and summarized based on algorithm type, annotations, diagnostic tests, and validation.

## 3. Results

### 3.1. Cohort 1—Demographics

Out of the 100 patients of Cohort 1, three CTs could not be processed by the algorithm due to high-grade osteopenia, and one CT did not show a fracture in retrospect. A total of *N* = 96 patients with *n* = 328 fractures of the cervical (*n* = 135), thoracic (*n* = 125), and lumbar (*n* = 68 lumbar) spine were analyzed. The cervical spine was affected in *n* = 60 cases, the thoracic and lumbar spine in *n* = 41 and *n* = 32 cases. The mean age was 59.4 ± 22.34 years (range 17–94 years) and 75% (*n* = 72) were male. Cervical and lumbar spine fractures were caused by falls in 65.3% and 73.7% of cases, whereas thoracic spine fractures were caused by falls in 46.4% of cases, but were also frequently associated with car (21.4%) and motorcycle accidents (14.3%) (Table S2).

### 3.2. Cohort 1—Analysis of Algorithm Version 1.0

Version 1.0 flagged 88 of 96 CT scans as conspicuous, resulting in a sensitivity per CT of 0.92 (95% CI 0.84–0.96). A total of 185 of 328 fractures were identified correctly (sensitivity per fracture 0.56, 95% CI 0.51–0.62). FP results occurred in 71.9% (69 of 96) of cases; in 39.6% of cases ≥ three FP results were recorded, in 18.8% one FP result, and two FP results in 16.7% cases.

When assessed separately for each spinal segment (Table 1), the cervical spine showed the highest sensitivity (0.87, 95% CI 0.76–0.93), followed by the lumbar spine (0.84, 95% CI 0.68–0.93) and the thoracic spine (0.68, 95% CI 0.53–0.80). Evaluation per fracture rather than per CT resulted in lower sensitivities primarily for the thoracic spine (0.34, 95% 0.26–0.42) and the lumbar spine (0.49, 95% CI 0.37–0.60), while the detection rate of cervical spine fractures remained comparatively high (sensitivity 0.82, 95% 0.74–0.87). Most FP results occurred in the lumbar spine, with 59.4% of cases with ≥three FP results and only 12.5% of cases without any FP results (two FP results in 9.4% cases, one FP result in 18.8%). In the thoracic spine, 51.2% of cases contained ≥three FP results, 4.9% contained two FP results, 19.5% of cases contained one FP, and 24.2% of cases contained none (Table S3).

A total of 45.8% (*n* = 44) of WBCTs were free of artifacts, whereas beam-hardening artifacts were present in 75% of FN cases and 59% of FP cases; artifacts caused by foreign bodies occurred in 63% of FN and 49% of FP cases. Motion artifacts were detected in only 12.5% (FN) and 17.4% (FP) of cases. The diverse reasons for false AI results are listed in Table 2. Of note, none of the false positive cases were attributed to pronounced trabecular structure.

### 3.3. Cohort 2—Demographics

Of 1074 eligible patients of the DOREMI trial, *n* = 712 WBCTs were randomly presented to the algorithm to fit the technical specifications of the hard- and software. Unsuccessful segmentation most likely related to severe osteopenia was observed in *n* = 48 cases, and in *n* = 1 case segmentation failed because of missing thin sliced WBCT data. In total, *n* = 663 were successfully segmented and analyzed. The mean age was 51.2 ± 18.69 years (range 8–92) and 66.1% (*n* = 438) of participants were male (Table S2). Most accidents were car accidents (24.0%) and falls (21.6%). The prevalence of fractures in the analyzed cohort (*n* = 663) was 44.3% (*n* = 294). A total of *n* = 363 fractures were detected: 18.4% (*n* = 122) of WBCTs showed fractures of the lumbar segment, 14.0% (*n* = 93) of the thoracic segment, 7.8% (*n* = 52) of the cervical spine, and 4.1% (*n* = 27) of the sacral segment. An occipital condyle fracture caused by a fall was present in one case. A total of 21.3% of the WBCTs showed fractures of vertebral bodies, 14.6% of transverse processes, 4.8% of articular processes, 4.7% of spinous processes, and 3.8% to the arches. The demographics of Cases 1–100 are shown in Table S2.

### 3.4. Cohort 2—Analysis of Algorithm Version 1.0 and 2.0 (Cases 1–100)

The sensitivity of v1 per WBCT was 0.87 (CI 0.72–0.96) and 0.97 (CI 0.86–1.00) for v2, with the highest values for the thoracic (v1) and lumbar spine (v2). Fractures of the sacral segment were detected with low sensitivity (Table 3).

Version 1.0 flagged 76 CTs as having at least one spinal fracture, with a total of 118 individual fracture detections (2.7 ± 3.01 [range 0–16]). Version 2.0 flagged 98 CTs and resulted in a total of 277 individual fracture detections (9.0 ± 5.15 [range 1–28]). Version 1.0 located most findings in the lumbar spine (55.1%), whereas Version 2.0 more frequently indicated spinal fractures in the cervical (33.9%) and thoracic (32.9%) spine. Table 4 describes the quality of the AI analysis in light of the number of FP per CT. Version 1.0 resulted in ≥ three FP findings in 35 cases, and Version 2.0 in 96 cases. The reasons for false AI results differed between the algorithms regarding FP findings, with most FPs caused by disks for v1 and by bony structures (trabecular structure, spondylophyts, bone canals) for v2 (Table S4; Figure S1).

### 3.5. Cohort 2—Analysis of Algorithm Version 2.0 (Cases 1–663)

The sensitivity of v2 was comparable for the cervical, thoracic, and lumbar spine, reaching 81–88% (Table 5). Sacral fracture detection again showed low sensitivity. The high number of detections per WBCT resulted in a sensitivity of 100%, with a high number of FPs per case. A varying ability of the algorithm in the detection of certain anatomical structures was not observed.

As expected per working setting, v2 resulted in ≥three FP findings in 97.1% (*n* = 644) of cases. TN and exclusively TP results both occurred in 0.2% (*n* = 1) of cases. WBCTs were flagged correctly (one TP plus FPs) in 30.0% (*n* = 199) of cases. Only 8.3% (*n* = 55) of WBCTs were free of artifacts, whereas 76.0% (*n* = 504) of WBCTs showed beam-hardening artifacts, 62.4% (*n* = 414) artifacts caused by foreign bodies, and 2.4% (*n* = 16) artifacts caused by motion. However, the portion of false AI analysis (FP, FN) was comparable in WBCTs with and without artifacts (Table S5). The reasons for false AI analysis in Cohort 2 overlapped with the results of Cohort 1 (Table 2); however, a prominent trabecular structure was suspected to cause most FPs in Cohort 2, followed by spondylophytes and bone canals.

### 3.6. Systematic Database Search

A total of 1255 sources were gathered and screened. Most articles were excluded since they described algorithms which detected compression fractures exclusively or algorithms focusing on either the cervical, thoracal, lumbar, or thoracolumbar spine. Five suitable articles describing convolutional neural networks (CNNs) published 2016–2025 were included in the systematic review. None of the published algorithms were tested on external datasets. Four algorithms were designed to detect fractures of the whole spine, and one algorithm was suitable for levels C3–L5. The reported metrics differed between studies. The sensitivities or recall rates of two algorithms varied between 71% and 81% for Roth et al. [17] and 59–90% for D.N. et al. [18]. The F1 scores ranged between 54 and 94% [19,20], and the Intersection over Union (IoU) ranged between 65 and 93% [19,21]. One study reported a precision of 69–75% [20], and two studies accuracies of 79–94% [18,20]. Specificities were not reported, but Roth et al. described false positive rates of 5 and 10 per patient [17].

## 4. Discussion

Our study aimed at expanding fracture detection towards comprehensive whole spine analysis. The data provided represent rare real-world results of an AI prototype for spine fracture detection on WBCTs. In a pilot study, we observed promising results of the network trained on cervical spines only, with good performance not only for the cervical but also for the thoracic spine due to generalization. Encouraged by the first results, further development and re-training of algorithm v2, including dedicated thoracic and lumbar spinal segments, produced rather decent results in a larger, more diverse cohort of WBCTs with a manifold set of fracture morphologies. While focusing on the ability to detect fractures in the first place, the false positives generated by v2 resulted in an inability to identify healthy cases. This may be largely attributed to the selected working settings which would be clinically intractable.

The reasons for the false AI results were manifold but overlapped in the two test cohorts. However, the comparison of v1 and v2 showed that further training seemed to result in a misinterpretation of the trabecular structure itself by a sensitized algorithm. Training data and Cohort 1 imaging was derived (with and without contrast enhancement) from a dual-source 2 × 192 row CT, while Cohort 2 was examined using a single-source 128 row scanner. Images of the cohorts differed in dose, resolution, and reconstruction mechanisms. From a developmental perspective, misinterpretation of the trabecular structure was potentially caused by domain shifts due to these variations in training and test data because neither version of the algorithm had been trained on data from the 128 row scanner. The mean ages of the primary training set (65 years), Cohort 1 (59 years), and Cohort 2 (51 years), as well as the gender distributions, also differed and might have additionally affected algorithm performance.

The study at hand has several limitations to be considered. The results of Cohort 1 describe a retrospective approach with highly selected cases positive for spine fractures. In this context, these results have a high risk for bias and need to be interpreted with caution. Considering the results of Cohort 2, which, in contrast, represent prospectively enrolled real-world cases, counterbalance our conclusions. Additionally, algorithm training was performed on monocentric data from a single CT scanner, which limited generalizability and potentially caused the above-described domain shift during analysis of Cohort 2. Furthermore, the reasons for false positive findings are presented semi-quantitively and their interpretation remains somewhat equivocal based on the algorithm’s output. Moreover, our aim to reach the highest sensitivity at a maximum of five false positives per case with the algorithm precluded reporting of its specificity as a comparable diagnostic parameter. The working settings within this ‘proof-of-concept’ approach are also responsible for the impracticality of the algorithm for clinical routine at this developmental stage. In its current form, the algorithm cannot identify individuals without spine fractures and prioritize those with critical findings, much less perform autonomous diagnosis. Implementation into clinical routine despite the current limitations would provoke serious ‘AI fatigue’ and lead to rejection bias in users.

Five articles which described similar algorithms were identified through a systematic literature search [17,18,19,20,21] (Table 6). The number of fractures used for algorithm training was similar in two studies [19,20], smaller in two other studies [17,21], and larger in one study [18]. The studies reported different, selected performance metrics and none of the research groups had tested their prototype on an independent dataset; hence, the results are hardly comparable. However, the ranges of accuracy of these algorithms were broad and the false positive rates, even though mentioned separately in only one instance (5 and 10 false positives per case; Ref. [17]), were presumably significant. All researchers worked on inherently different cohorts from various countries which impedes balanced comparisons, i.e., Zhang et al. [20] examined a younger cohort compared to our data. Still, the reported performance metrics in these studies roughly attain the results of v1 of our algorithm regarding sensitivity. The ground truth used to train v2 of our prototype was likely too diverse, with insufficient representation and improper balancing of different fracture types and locations. This is especially important because cervical fractures were used most frequently during training but represented the lowest prevalence in Cohort 2. Consequently, the training sets turned out to be unrepresentative for this real-world data. Further development with increased training data and refined calibration of the algorithm might improve its specificity towards clinical applicability. We can only speculate why the published algorithms were not further evaluated and developed for an intended clinical use, but a high false positive rate seems plausible as one relevant factor.

As annotations of data and profound training of algorithms are demanding tasks, in essence, five major factors complicated the development of a comprehensive spine fracture detection tool for WBCTs: the rarity of certain fracture types (particularly of the posterior elements), the diversity of fractures, the wide variety of anatomical variants, varying degrees of degenerative processes, artifacts, and insufficient quantity of thoracic and lumbar training cases. Care has to be taken for proper cohort design to ensure that an algorithm is able to generalize well. Other groups also indicated that fracture detection is essentially feasible but challenging due to heterogenous fractures and the presence of artifacts [17,19,20].

In summary, it is technically feasible to detect all types of individual spine fractures on WBCT with the described algorithm. The high false positive rate and thus its inability to identify healthy cases impede clinical utility at the moment. Multiple factors contribute to the difficulties of developing a commercially available AI tool for comprehensive spine fracture detection on WBCT for severely injured patients. The rarity and diversity of spine fractures, anatomical variants, and degenerative processes alike limit generalizability during training, while imaging artifacts and varying CT imaging parameters cause domain shifts which are also deemed responsible for this challenging task. Current progress in AI research gives reason for hope that new techniques will be able to overcome these challenges sooner than expected. However, efforts must be focused on expanding and designing proper training cohorts representing real-life conditions. Multi-site data for algorithm training is desirable to cover diversity issues and collect enough fracture morphologies and CT image qualities. Data augmentation methods have been successfully used by us and other groups [18,21], and increased training and test cohorts will additionally be used. Transfer learning, semi-supervised training techniques, implementation of efficient annotation strategies, federated learning, few-shot learning, and alternative neural network architectures such as transformer models [22] are promising approaches of upcoming algorithm development. Finally, synthetic data simulating artificial CT images with spine fractures also address the shortage of high-quality data [23,24], and could prove potent in enhancing the development of WBCT spine analysis in the near future as well.

## Figures and Tables

**Table 1:** Diagnostic Accuracy in Population 1 per CT and per Fracture (Prototype 1).

Per CT		TP	FN	Sensitivity (95% CI)
Cervical (*n* = 60)	*n*	52	8	0.87 (0.76–0.93)
Thoracic (*n* = 41)	*n*	28	13	0.68 (0.53–0.80)
Lumbar (*n* = 32)	*n*	27	5	0.84 (0.68–0.93)
Per Segment				
Cervical (*n* = 135)	*n*	110	25	0.82 (0.74–0.87)
Thoracic (*n* = 125)	*n*	42	83	0.34 (0.26–0.42)
Lumbar (*n* = 68)	*n*	33	35	0.49 (0.37–0.60)

**Table 2:** Reasons for False AI Results in Populations 1 and 2.

	Population 1 *N* = 96	Population 2 *N* = 663		Population 1 *N* = 96	Population 2 *N* = 663
FP findings *n* (%)			FN findings *n* (%)		
Spondylophyte	38 (55.1)	394 (59.4)	Close proximity to another fracture/finding	11 (21.6)	18 (2.7)
Calcification of ligament	13 (18.8)	35 (5.3)	Osteopenia	3 (5.9)	2 (0.3)
Contrast agent in veins	11 (15.9)	142 (21.4)	Adjacent FP finding	1 (2.0)	10 (1.5)
Bone canal	26 (37.7)	350 (52.8)	Discrete compression fracture	20 (39.2)	12 (1.8)
Motion artifact	5 (7.2)	13 (2.0)	No dislocation	18 (35.3)	82 (12.4)
Disk	31 (44.9)	14 (2.1)	Adjacent spondylophyte flagged	3 (5.9)	1 (0.2)
Calcified disk	8 (11.6)	7 (1.1)	Luxation of facet joint	3 (5.9)	7 (1.1)
Facet joint space	14 (20.3)	90 (13.6)	Close proximity to degenerative structure	3 (5.9)	6 (0.9)
Osteoarthritis of facet joint	5 (7.2)	102 (15.4)	Extremely dislocated fracture	-	2 (0.3)
Anatomical variant	1 (1.4)	24 (3.6)	Motion artifact	3 (5.9)	
Bone metastasis	2 (2.9)	1 (0.2)	Disk flagged	3 (5.9)	
Joint space	4 (5.8)	28 (4.2)	Old fracture	-	8 (1.2)
Schmorl’s nodes	2 (2.9)	62 (9.4)	No apparent reason	3 (5.9)	3 (0.5)
Costovertebral joint	1 (1.4)	21 (3.2)			
Prominent trabecular structure	-	539 (81.3)			
Rib fracture	-	6 (0.9)			
Foreign body	-	4 (0.6)			

**Table 3:** Diagnostic Accuracy and Performance Metrics of both Prototype Versions per Segment and CT (Population 2 Cases 1–100).

Segment	Version	TP	FN	TN	FP	Sensitivity (95% CI)
Cervical (*n* = 13)	v1	8	5	72	15	0.62 (0.32–0.86)
	v2	10	3	6	81	0.77 (0.46–0.95)
Thoracic (*n* = 13)	v1	9	4	70	17	0.69 (0.39–0.91)
	v2	10	3	7	80	0.77 (0.46–0.95)
Lumbar (*n* = 20)	v1	12	8	32	48	0.60 (0.36–0.81)
	v2	17	3	25	55	0.85 (0.62–0.97)
Sacral (*n* = 5)	v1	1	4	94	1	0.20 (0.05–0.72)
	v2	2	3	78	17	0.40 (0.05–0.85)
Per WBCT (*n* = 100)	v1	33	5	19	43	0.87 (0.72–0.96)
	v2	37	1	1	61	0.97 (0.86–1.00)

TP—True True positive, FN—false False negative, TN—true True negative, FP—false False positive, CI—confidence interval, WBCT—whole-body CT.

**Table 4:** AI Analysis Results of Population 2: Version 1.0 vs. Version 2.0 (Cases 1–100).

	V 1.0 (*N* = 100)	V 2.0 (*N* = 100)		V 1.0 (*N* = 100)	V 2.0 (*N* = 100)
Number of annotations, mean ± SD [Range]	2.7 ± 3.01 [0–16]	9.0 ± 5.15 [1–28]	Quality of AI analysis, *n* (%)	2	1
CT flagged, *n* (%)			Exclusively TP	19	0
Yes	76	98	1 FP	19	2
No	21	2	2 FP	35	96
Missing	2	-	≥3 FP	30	23
Fracture location according to AI, *n* (%)			FN	26	34
Cervical	23	94	1 TP (plus FPs)	17	0
Thoracic	28	91	TN	2	1
Lumbar	65	73			
Sacral	2	19			

SD—Standard deviation, FP—false positive, FN—false negative, TP—true positive, TN—true negative.

**Table 5:** Diagnostic Accuracy and Performance Metrics of Prototype 2 Per Segment and CT (Population 2, *N* = 663).

Segment	TP	FN	TN	FP	Sensitivity (95% CI)
Cervical (*n* = 52)	42	10	87	524	0.81 (0.67–0.90)
Thoracic (*n* = 93)	75	18	31	539	0.81 (0.71–0.88)
Lumbar (*n* = 122)	107	15	143	398	0.88 (0.81–0.93)
Sacral (*n* = 27)	12	15	545	91	0.44 (0.26–0.65)
Per WBCT	222	1	1	436	1.00 (0.98–1.00)

TP—True positive, FN—false negative, TN—true negative, FP—false positive, CI—confidence interval, WBCT—whole-body CT.

**Table 6:** Systematic Review Results.

Author	Country	Number of CT Scans/ Fractures	Anatomical Region	Algorithm Type	Ground Truth/Annotations	Diagnostic Test/ Analysis Related Variables	Validation (Internal and External)
Roth et al. [17] 2016	USA	23/55	Posterior elements, whole spine	CNN	Radiologists	Sensitivity 71/81% 5/10 false positives per patient	No, training and testing dataset
D.N. et al. [18] 2025	India	ns/2820	C3–L5	CNN	Spinal surgeons	Sensitivity 59–90%, F1 score 54–94%; accuracy 89.98–93.68%	No, training and testing dataset
Saeed et al. [19] 2024	China/ Saudi Arabia	235/ns	Whole spine	CNN	N/s	F1 score 78–92%, Intersection over Union 80–93%	No, training, validation, and testing dataset
Zhang et al. [20] 2023	China	197/311	Whole spine, three columns	CNN	Radiology reports, no expert annotations	F1 score 69–78%, accuracy 79–88%	No, training, validation, and testing dataset
Sha et al. [21] 2021	China	/40	Whole spine	CNN	No expert annotations	Precision 69–75%, Intersection over Union 65–76%	No, training, validation, and testing dataset

## Data Availability

The original contributions presented in this study are included in the article/Supplementary Material. Deidentified participant data are available at the German Clinical Trial Registry for DRKS00029356 (https://www.bfarm.de/DE/Das-BfArM/Aufgaben/Deutsches-Register-Klinischer-Studien/_node.html) (accessed on 26 May 2026). Further inquiries can be directed to the corresponding author.

## Supplementary Materials

The following supporting information can be downloaded at: https://www.mdpi.com/article/10.3390/jimaging12060265/s1, Figure S1: False positive analysis by the prototype v2 showing a highlighted bone canal (blue) and endplate (lilac) (A: coronal, B: sagittal). False positive analysis attributed to metal artifacts resulting in a highlighted spinal process (green) and anterior margin of the vertebrae (blue) (C: coronal, D: sagittal); Table S1: Search Strategies; Table S2: Demographics of populations 1 and 2; Table S3: False positive results; Table S4: Reasons for false AI results in population 2 cases 1–100; Table S5: Influence of artifacts on AI analysis in population 1 and 2.

## Abbreviations

ED	emergency department
WBCT	whole-body computed tomography
AI	artificial intelligence
v1	version 1.0
v2	version 2.0
TP	true positive
TN	true negative
FP	false positive
FN	false negative
CIs	confidence intervals
SD	standard deviation
CNN	convolutional neural networks
IoU	Intersection over Union
