# Supplementary material for: Towards Automated Spine Fracture Detection on Whole-Body CT of Polytraumatized Patients
Source: J Imaging. 2026 Jun 18;12(6):265. doi: 10.3390/jimaging12060265 (PMC13300958; doi:10.3390/jimaging12060265)
Supplement: Supplementary file 1 [file jimaging-12-00265-s001.zip › Supplement.pdf]

SUPPLEMENT

Table S1. Search Strategies.

**Database**	**Date**	**Search strategy**
PubMed (MEDLINE)	Feb. 7th, 2026	("Spinal Fractures"[Mesh] OR "spinal fracture"[Title/Abstract] OR "spinal fractures"[Title/Abstract] OR "vertebral fracture"[Title/Abstract] OR "vertebral fractures"[Title/Abstract] OR "spine fracture"[Title/Abstract] OR "spine fractures"[Title/Abstract]) AND ("Artificial Intelligence"[Mesh] OR "Machine Learning"[Mesh] OR "Deep Learning"[Mesh] OR "Neural Networks, Computer"[Mesh] OR "machine learning"[Title/Abstract] OR "deep learning"[Title/Abstract] OR "automated detection"[Title/Abstract] OR "artificial intelligence"[Title/Abstract] OR AI[Title/Abstract] OR cnn[Title/Abstract] OR "convolutional neural network"[Title/Abstract] OR "convolutional neural networks"[Title/Abstract]) AND (english[lang]) AND ("2015"[Date - Publication] : "3000"[Date - Publication])
Embase (Ovid/Elsevier)	Feb. 7th, 2026	('spinal fracture'/exp OR 'spine fracture':ti,ab OR 'vertebral fracture':ti,ab OR 'spinal fractures':ti,ab) AND ('artificial intelligence'/exp OR 'machine learning'/exp OR 'deep learning'/exp OR 'neural network'/exp OR 'machine learning':ti,ab OR 'deep learning':ti,ab OR 'automated detection':ti,ab OR 'artificial intelligence':ti,ab OR cnn:ti,ab OR 'convolutional neural network':ti,ab) AND [english]/lim AND [2015-2025]/py
Web of Science (Core Collection)	Feb. 7th, 2026	TS=("spinal fracture" OR "spinal fractures" OR "vertebral fracture" OR "vertebral fractures" OR "spine fracture" OR "spine fractures") AND TS=("machine learning" OR "deep learning" OR "automated detection" OR "artificial intelligence" OR cnn OR "convolutional neural network") Refined by: LANGUAGES: (ENGLISH) AND PUBLICATION YEARS: (2015-2025)
Cochrane Library	Feb. 7th, 2026	([mh "Spinal Fractures"] OR "spinal fracture":ti,ab,kw OR "spinal fractures":ti,ab,kw OR "vertebral fracture":ti,ab,kw) AND ([mh "Artificial Intelligence"] OR [mh "Machine Learning"] OR [mh "Deep Learning"] OR "machine learning":ti,ab,kw OR "deep learning":ti,ab,kw OR "automated detection":ti,ab,kw OR "artificial intelligence":ti,ab,kw OR cnn:ti,ab,kw OR "convolutional neural network":ti,ab,kw)

Table S2. Demographics of populations 1 and 2.

	Population 1			Population 2							
	N=96			N=663				N=Cases 1-100			
Gender, n (%)	cervical (N=49)	thoracic (N=28)	lumbar (N=19)	cervical (N=52)	thoracic (N=93)	lumbar (N=122)	sacral (N=27)	cervical (N=13)	thoracic (N=13)	lumbar (N=20)	sacral (N=5)
Male	72 (75.0)			438 (66.1)				70 (70.0)			
Female	24 (25.0)			223 (33.6)				30 (30.0)			
Missing	-			2 (0.3)				-			
Age, mean ± SD [range]	59.4 ± 22.34 [17-94]			51.2 ± 18.69 [8-92]				48.4 ± 18.60 [8-92]			
Mechanism											
Fall	32 (65.3)	13 (46.4)	14 (73.7)	25 (48.1)	53 (57.0)	62 (50.8)	13 (48.1)	7 (53.8)	4 (30.8)	10 (50.0)	4 (80.0)
Car accident	6 (12.2)	6 (21.4)	3 (15.8)	14 (26.9)	17 (18.3))	29 (23.8))	8 (29.6)	2 (15.4))	3 (23.1)	3 (15.0)	-
Bike accident	3 (6.1)	-	1 (5.3)	4 (7.7)	5 (5.1)	5 (4.1)	3 (11.1)	1 (7.7)	2 (15.4)	1 (5.0)	-
Pedestrian vs. car	3 (6.1)	-	-	1 (1.9))	1 (1.1)	4 (3.3)	-	-	1 (7.7)	1 (5.0)	-
Motorcycle accident	2 (4.1)	4 (14.3)	-	2 (3.8)	8 (8.6)	9 (7.4)	1 (3.7)	1 (7.7)	2 (15.4)	3 (15.0)	-
Head dive	-	2 (7.1)	-	2 (3.8)	1 (1.1)	-	-	-	-	-	-
Uncertain	3 (6.1)	3 (10.7)	1 (5.3)	3 (5.8))	3 (3.2)	4 (3.3)	2 (7.4)	2 (15.4)	1 (7.7)	1 (5.0)	1 (20.0)
Explosion/Fire	-	-	-	-	2 (2.2)	2 (1.6)	-	-	-	-	-
Unspecified blunt trauma	-	-	-	1 (1.9)	3 (3.2)	5 (4.1)	-	-	-	-	-
Assault	-	-	-	-	-	1 (0.8)	-	-	-	-	-
Train accident	-	-	-	-	-	1 (0.8)	-	-	-	1 (5.0)	-

SD – standard deviation

Table S3. False positive Results.

		Population 1 (N=96)				Population 2 (N=663)			
		0	1	2	>=3	0	1	2	>=3
Per WBCT	n/N	27/96	18/96	13/96	38/96	5/663	5/663	9/663	644/663
	%	28.13	18.75	16.67	39.58	0.75	0.75	1.36	97.13
Per segment						N/A			
cervical	n/N	14/49	13/49	9/49	13/49				
	%	28.57	26.53	18.37	26.53				
thoracic	n/N	10/28	2/28	1/28	15/28				
	%	35.71	7.14	3.57	53.57				
lumbar	n/N	3/19	3/19	3/19	10/19				
	%	15.79	15.79	15.79	52.63				

N/A – not analyzed

Table S4. Reasons for false AI results in population 2 cases 1-100.

	V 1 (N=100)	V2 (N=100)
**FP findings**		
Spondylophyt	18	49
Calcification of ligament	6	10
Contrast agent in veins	5	26
Bone canal	14	52
Motion artifact	2	2
Disc	53	5
Calcified disc	2	2
Facet joint space	4	13
Osteoarthritis of facet joint	3	13
Anatomical variant	3	5
Joint space	1	10
Schmorl´s nodes	8	15
Costovertebral joint	0	5
Prominent trabecular structure	2	59
Rib fracture	0	3
**FN findings**		
Close proximity to another fracture/finding	4	3
Motion artifact	0	0
Osteopenia	1	2
Discrete compression fracture	5	2

no dislocation	20	13
Adjacent spondylophyt flagged	0	0
luxation of facet joint	3	3
Close proximity to degenerative structure	2	2
Adjacent disc flagged	3	0
Extremely dislocated fracture	0	1
Old fracture	0	1
No apparent reason	2	2

FP – false positive, FN – false negative

Table S5. Influence of Artifacts on AI analysis in Population 1 and 2.				
	Population 1		Population 2	
	≠ artifacts (N=28)	artifacts (N=68)	≠ artifacts (N=55)	artifacts (N=608)
FP, n (%)				
cervical	6 (21.4)	29 (42.6)	45 (81.8)	479 (78.8)
thoracic	4 (14.3)	14 (20.6)	44 (80.0)	495 (81.4)
lumbar	6 (21.4)	8 (11.8)	29 (52.7)	369 (60.7)
sacral			9 (16.3)	82 (13.5)
FN, n (%)				
cervical	1 (0.4)	7 (10.3)	1 (1.8)	9 (1.5)
thoracic	3	10 (14.7)	1 (1.8)	17 (2.8)
lumbar	1 (10.7)	4 (5.9)	-	15 (2.5)
sacral			1 (1.8)	14 (2.3)

FP – false positive, FN – false negative

Supplement Figure Legend:

Figure S1: False positive analysis by the prototype v2 showing a highlighted bone canal (blue) and endplate (lilac) (A: coronal, B: sagittal). False positive analysis attributed to metal artifacts resulting in a highlighted spinal process (green) and anterior margin of the vertebrae (blue) (C: coronal, D: sagittal).
