# Supplementary figures and images for: Towards Automated Spine Fracture Detection on Whole-Body CT of Polytraumatized Patients
Source: J Imaging. 2026 Jun 18;12(6):265. doi: 10.3390/jimaging12060265 (PMC13300958; doi:10.3390/jimaging12060265)

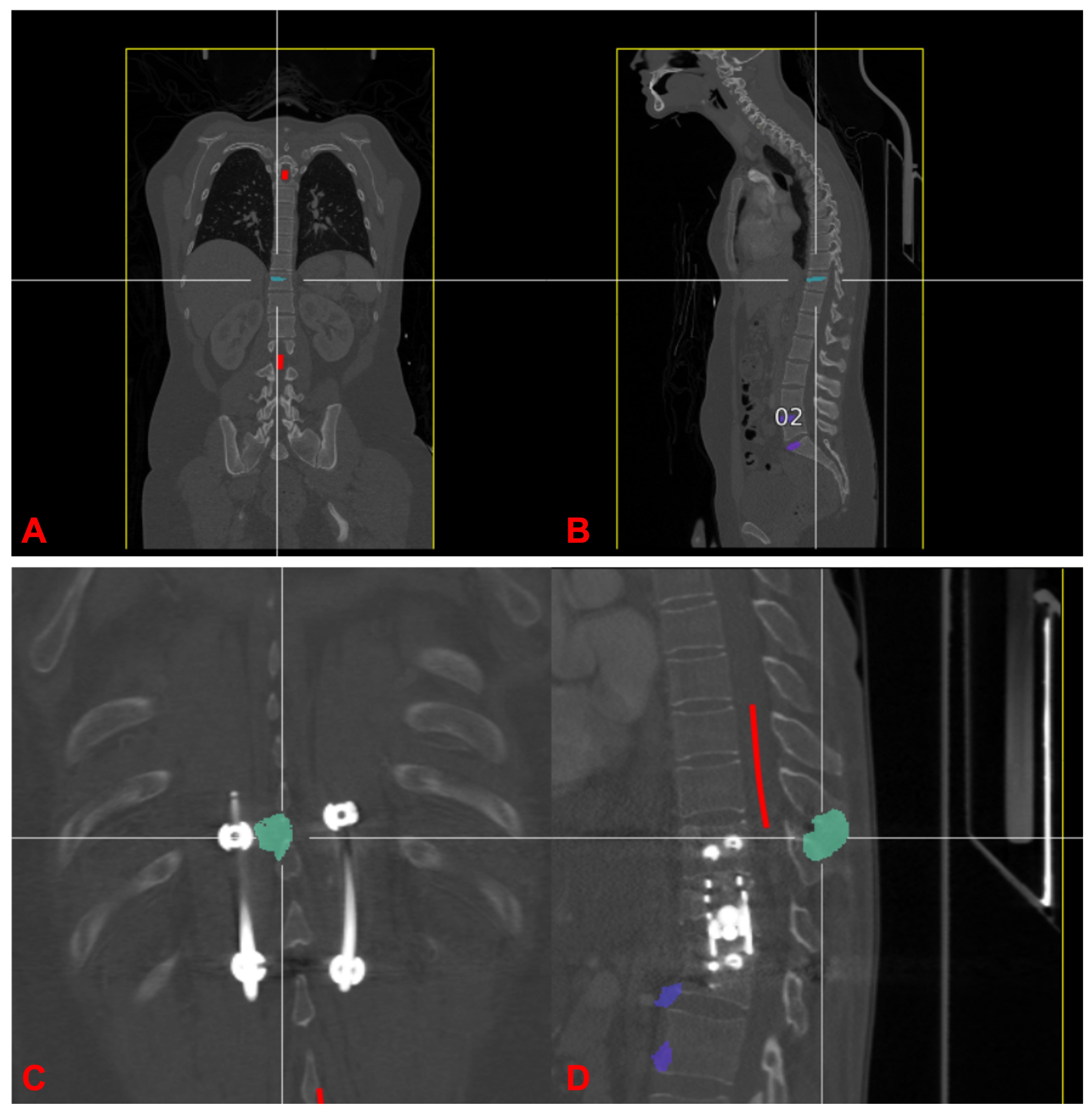

Supplement: Supplementary file 1 [file jimaging-12-00265-s001.zip › S1.jpg]
